# Association of Tim-3/Gal-9 Axis with NLRC4 Inflammasome in Glioma Malignancy: Tim-3/Gal-9 Induce the NLRC4 Inflammasome

**DOI:** 10.3390/ijms23042028

**Published:** 2022-02-12

**Authors:** JeongMin Sim, JeongMan Park, Suwan Kim, Sojung Hwang, KyoungSu Sung, Jung-Eun Lee, SeungHo Yang, Kyunggi Cho, SungHwan Lee, Jong-Seok Moon, JuWon Ahn, Jaejoon Lim

**Affiliations:** 1 Department of Neurosurgery, Bundang CHA Medical Center, CHA University, Seongnam 13496, Korea; simti123@chauniv.ac.kr (J.S.); jungman.park@chauniv.ac.kr (J.P.); suwankimm@chauniv.ac.kr (S.K.); sjhwang7@chamc.co.kr (S.H.); sandori50@chamc.co.kr (K.C.); 2 Department of Biomedical Science, College of Life Science, CHA University, Seongnam 13488, Korea; 3 Global Research Supporting Center, Bundang CHA Medical Center, CHA University College of Medicine, Seongnam 13496, Korea; 4 Department of Neurosurgery, Dong-A University Hospital, Dong-A University College of Medicine, Busan 49201, Korea; sungks@dau.ac.kr; 5 Department of Neurosurgery, St. Vincent’s Hospital, College of Medicine, The Catholic University of Korea, Seoul 16247, Korea; eunree@catholic.ac.kr (J.-E.L.); 72ysh@catholic.ac.kr (S.Y.); 6 Department of Surgery, Bundang CHA Medical Center, CHA University, Seongnam 13496, Korea; leeshmd77@cha.ac.kr; 7 Soonchunhyang Institution of Medi-Bio Science (SIMS), Soonchunhyang University, Cheonan-si 31151, Korea; jongseok81@sch.ac.kr

**Keywords:** glioma, glioma malignancy, NLRC4 inflammasome, Tim-3/Gal-9

## Abstract

Tim-3/Gal-9 and the NLRC4 inflammasome contribute to glioma progression. However, the underlying mechanisms involved are unclear. Here, we observed that Tim-3/Gal-9 expression increased with glioma malignancy and found that Tim-3/Gal-9 regulate NLRC4 inflammasome formation and activation. Tim-3/Gal-9 and NLRC4 inflammasome-related molecule expression levels increased with WHO glioma grade, and this association was correlated with low survival. We investigated NLRC4 inflammasome formation by genetically regulating Tim-3 and its ligand Gal-9. Tim-3/Gal-9 regulation was positively correlated with the NLRC4 inflammasome, NLRC4, and caspase-1 expression. Tim-3/Gal-9 did not trigger IL-1β secretion but were strongly positively correlated with caspase-1 activity as they induced programmed cell death in glioma cells. A protein–protein interaction analysis revealed that the FYN-JAK1-ZNF384 pathways are bridges in NLRC4 inflammasome regulation by Tim-3/Gal-9. The present study showed that Tim-3/Gal-9 are associated with poor prognosis in glioma patients and induce NLRC4 inflammasome formation and activation. We proposed that a Tim-3/Gal-9 blockade could be beneficial in glioma therapy as it would reduce the inflammatory microenvironment by downregulating the NLRC4 inflammasome.

## 1. Introduction

Gliomas are the most common types of malignant brain tumor [1]. The inflammatory microenvironment increases during glioma progression and promotes tumor development and chemoresistance [2,3]. Consequently, it is difficult to establish efficacious therapeutic strategies such as surgery, radiotherapy, chemotherapy, or immunotherapy for gliomas [4]. Recent studies have furnished empirical and clinical evidence that the inflammatory microenvironment and immune evasion are hallmarks of glioma progression [5,6,7]. In many tumors, these mechanisms do not act alone. Rather, they are interconnected and collaborate in malignant tumor transformation [8,9,10]. Hence, understanding the relationships among mechanisms associated with glioma development is vital to establishing novel diagnostic and treatment approaches.

Immune system dysfunction is a crucial factor in glioma carcinogenesis [11]. During glioma development, tumor cells upregulate immune checkpoint molecules and their ligands. In this manner, they induce a microenvironment in the surrounding cells in which the tumor can thrive and evade host immunity [5,12]. Tim-3 (T cell immunoglobulin and mucin-domain containing-3) is a representative immune checkpoint molecule that is highly expressed in glioma [13,14,15]. It is activated primarily by its ligand Gal-9 (Galectin-9). Binding between Tim-3 and Gal-9 promotes tumor growth and immune escape in the tumor microenvironment (TME) [16]. Recent studies have demonstrated that Tim-3/Gal-9 regulate the inflammatory microenvironment by modulating inflammasome assembly and activation [17,18]. In vivo and ex vivo experiments showed that the roles of Tim-3/Gal-9 vary with inflammasome type. Tim-3/Gal-9 may be upstream from the inflammasome [18,19,20]. We previously reported that the NLRC4 inflammasome occurs in the glioma TME and is related to poor survival [21].

It was established that the inflammatory response in the TME plays a pivotal role in glioma progression [22]. In many tumors including gliomas, inflammasomes trigger the inflammatory response [23]. Inflammasomes constitute an innate immune system consisting of a NOD-like receptor (NLR), adapter apoptosis-associated speck like protein (ASC), and pro-caspase-1 [24]. They detect infectious microorganisms and their proteins, as well as pathogen-associated molecular patterns (PAMPs), danger-associated molecular patterns (DAMPs), reactive oxygen species (ROS), ATP, and DNA. They induce the inflammatory response by activating caspase-1 and stimulating IL-1β and IL-18 proinflammatory cytokine secretion [25]. Inflammasome oligomerization induces pyroptosis, which is an inflammatory programmed cell death response to intracellular pathogens [26]. We previously verified that NLRC4 inflammasome expression significantly increases with glioma progression [21]. Tim-3/Gal-9 can activate the NLRC4 inflammasome [27]. There is a clinical correlation between the immunomodulatory role of Tim-3/Gal-9 and the NLRC4 inflammasome. Hence, it is essential to elucidate the molecular mechanism underlying the actions of Tim-3/Gal-9 and their interactions with the NLRC4 inflammasome.

In this study, we investigated the molecular connections between Tim-3/Gal-9 and the NLRC4 inflammasome in glioma. We determined that Tim-3/Gal-9 regulate NLRC4 inflammasome assembly and activation by upregulating NLRC4 and caspase-1 and modulating programmed cell death.

## 2. Methods

### 2.1. Description of TCGA Data

RNA-seq, clinical, and survival data for GBM and LGG were downloaded from the UCSC XENA database (https://xena.ucsc.edu/, accessed on 8 September 2021). The GBMLGG dataset comprised 537 primary samples including astrocytoma, oligodendroglioma, and glioblastoma. All samples had clinical, survival, and RNA-seq information. A comparison of gene expression between normal tissue and tumors was conducted with the TCGA-TARGET-GTEx dataset in UCSC XENA, and 1152 samples from normal brain tissue were used.

### 2.2. Correlation Analysis and Correlation Coefficient Hierarchical Clustering

A correlation analysis was conducted to detect genes correlated with Gal-9 and Tim-3 expression. Intersecting genes were selected and visualized with Venny v. 2.1.0 (https://bioinfogp.cnb.csic.es/tools/venny/index.html, accessed on 8 September 2021). Correlation coefficient hierarchical clustering was conducted with heatmaply v. 1.3.0 (https://cran.r-project.org/web/packages/heatmaply/index.html, accessed on 8 September 2021) to identify genes highly correlated with Gal-9 and Tim-3.

### 2.3. Gene Ontology Enrichment Analysis

GO biological process and cellular component enrichment analyses were performed using ShinyGo v. 0.74 (http://bioinformatics.sdstate.edu/go/ accessed on 18 November 2021). Significantly enriched pathways with Benjamini–Hochberg correction <0.05 were extracted. The *p*-values of the enriched pathways were −log_10_-transformed.

### 2.4. Survival Analysis

A pairwise log rank test was conducted and a Kaplan–Meier plot was generated to compare prognosis among groups segregated by gene expression level. A survival analysis was conducted with Survival v. 3.2 (https://cran.r-project.org/web/packages/survival/index.html, accessed on 8 September 2021). The survival plot was visualized with Survminer v. 0.4.8 (https://cran.r-project.org/web/packages/survminer/index.html, accessed on 8 September 2021).

### 2.5. Patient Samples

Tissues were obtained from glioma patients at the CHA Bundang Medical Center, Republic of Korea, under Institutional Review Board approval (No. CHAMC2021-01-024-001). Written consent was secured from all donors and clinical information for patients was described in Table S1. Tissues were acquired during surgery at the Department of Neurosurgery of the CHA Bundang Medical Center of CHA University, Republic of Korea. They were stored at −80 °C.

### 2.6. Glioma Cells

A172, U373MG, and U87MG glioma cell lines expressing Tim-3 or Gal-9 were examined with DepMap (The Cancer Dependency Map Project at Broad Institute; https://depmap.org/portal/, accessed on 12 June 2021). They were procured from the Korean Cell Line Band (KCLB; Republic of Korea) and confirmed to be mycoplasma-free. They were grown in Dulbecco’s modified Eagle’s medium (DMEM; Thermo Fisher Scientific, Waltham, MA, USA) supplemented with 12.5% (*v/v*) fetal bovine serum (FBS; Thermo Fisher Scientific) and 1% (*v/v*) penicillin/streptomycin (Thermo Fisher Scientific) at 37 °C and under a 5% CO_2_ atmosphere.

Primary glioma cells were obtained from the histological grade 2, 3, and 4 tissues of glioma patients. They were mechanically sectioned into small pieces and enzymatically dissociated with collagenase l (Roche Diagnostics, Basel, Switzerland) and DNase l (Roche Diagnostics) at 37 °C for 30 min. The cells were washed with phosphate-buffered saline (PBS; Thermo Fisher Scientific) and enumerated with hemocytometer (Thermo Fisher Scientific). Then, cells at a density of 1.0 × 10^6^/mL were cultivated in DMEM supplemented with 12.5% FBS, 1% (*v/v*) penicillin/streptomycin, 50 ng/mL epidermal growth factor (EGF; Thermo Fisher Scientific), and 50 ng/uL fibroblast growth factor (FGF; PeproTech, Cranbury, NJ, USA) at 37 °C and under a 5% CO_2_ atmosphere. All media and reagents were lipopolysaccharide (LPS)-free. The FBS was heat-inactivated and contained <5 EU/mL endotoxin.

### 2.7. Transfection

Negative control (NC), si-Tim-3, si-Gal-9, pc-Tim-3, and pc-Gal-9 were prepared to study Tim-3 and Gal-9 functions in glioma. The sequences of the purchased siRNA and pcDNA are listed in Table S2. The glioma cells were incubated in six-well plates at a density of 1 × 10^6^/well for 20–24 h. Two hours before transfection, glioma cells at 80–90% confluence were incubated with fresh medium free of serum and antibiotics. Transfection was performed with Lipofectamine^TM^ RNAi MAX (Thermo Fisher Scientific) or Lipofectamine^TM^ 3000 (Thermo Fisher Scientific) according to the manufacturer’s instructions.

### 2.8. Immunohistochemistry

All glioma patient tissues, including grade 2, 3, and 4, used in IHC were obtained surgically, and the number of patients and pathological information were recorded in Supplementary Table S1. Formalin-fixed, paraffin-embedded glioma blocks were serially sectioned at 4 µm thickness and deparaffinized in xylene (Thermo Fisher Scientific). The sections were immersed in ethanol, rehydrated, and incubated with goat serum to block nonspecific binding. They were then incubated at 4 °C overnight with primary antibodies against Gal-9 (mouse monoclonal Ab; Abcam, Cambridge, UK), TIM-3 (rabbit monoclonal Ab; Abcam), GFAP (rabbit monoclonal Ab; Abcam), Iba-1 (rabbit monoclonal Ab; Abcam), NLRC4 (rabbit polyclonal Ab; Abcam), or caspase-1 (mouse monoclonal Ab; CASP-1; Santa Cruz Biotechnology, Dallas, TX, USA). Secondary antibodies, including goat anti-rabbit IgG (Abcam) and donkey anti-mouse IgG (Abcam), were applied at room temperature for 1 h. The sections were examined under a confocal laser scanning microscope (Zeiss LSM; Carl Zeiss AG, Jena, Germany) and quantified using the ImageJ software v1.52a (NIH, Bethesda, MD, USA).

### 2.9. Flow Cytometry

Glioma cells were analyzed by flow cytometry to identify Tim-3, Gal-9, and NLRC4 inflammasome-related molecules on their surfaces. The Fc receptor was blocked with human Fc blocker (BD Biosciences, San Jose, CA, USA). The glioma cell lines were then stained with the antibodies listed in Table S3.

The expression levels of intracellular molecules including Iba1, GFAP, and caspase-1 were determined by intracellular staining. Si-Tim-3 or si-Gal-9 was prepared and the cells were collected on day 3 and treated with permeabilization solution (BD Biosciences) at room temperature for 20 min. The cells were then washed with Perm/Wash Buffer (BD Biosciences) and stained at 4 °C for 30 min with the antibodies listed in Table S3. To exclude dead cells from the flow cytometry data, the cells were stained with 7-amino-actinomycin D (7-AAD; BD Pharmingen, San Diego, CA, USA) at a concentration of 5 μL/10^6^ cells). The 7-AAD-negative glioma cell populations were analyzed. CytoFLEX (Beckman Coulter Inc., Brea, CA, USA) was used for all flow cytometry assays. All data were analyzed with FlowJo v. 10 (FlowJo LLC, Ashland, OR, USA).

### 2.10. TUNEL Assay

To evaluate in situ glioma cell death, 4 µm sections of formalin-fixed, paraffin-embedded blocks of glioma grades 2, 3, and 4 tissues were prepared. The sections were permeabilized with 0.1% (*w/v*) Triton X-100 and incubated with a terminal deoxynucleotidyl transferase dUTP nick-end labeling (TUNEL) reagent (Merck GmbH, Darmstadt, Germany). The stained samples were then treated with 4′,6-diamidino-2-phenylindole (DAPI) for 30 min. Apoptosis of single cells was examined under a confocal laser scanning microscope (Carl Zeiss AG, Jena, Germany).

### 2.11. FLICA^caspase−1^ Assay

G2, G3, and G4 primary glioma cells (1.0 × 10^6^ cell/mL) were grown in DMEM supplemented with 12.5% FBS, 1% (*v/v*) penicillin/streptomycin, 50 ng/mL EGF (Thermo Fisher Scientific), and 50 ng/µL FGF (PeproTech) at 37 °C for 3 day. To detect active caspase-1, 1.0 × 10^6^ glioma cells were prepared and treated with cell permeant FLICA 660-VAD-FMK (Neuromics, Minneapolis, MN, USA). After incubation at 37 °C for 4 h, cells were washed with fresh media and analyzed with flow cytometry.

### 2.12. Programmed Cell Death Analysis

To measure apoptosis resulting from Tim-3 or Gal-9 knockdown, glioma cells were placed in 0.1% (*v/v*) bovine serum albumin (BSA) and stained with Annexin V (BD Biosciences). The cells were then washed with Annexin V buffer (BD Biosciences) and stained with 7-AAD (BD Pharmingen). To exclude spontaneous cell death, the % apoptosis was calculated based on the Annexin V and 7-AAD signal generated by control living glioma cells that were not treated with siRNA. Apoptosis was quantitated by flow cytometry (CytoFLEX; Beckman Coulter).

### 2.13. ELISA

Glioma cells were cultured in six-well plates at a density of 6 × 10^5^/well. They were incubated for 2 d in the presence of si-Tim-3 or si-Gal-9 and subjected to flow cytometry and qRT-PCR. Supernatant from the culture medium was collected and stored at −80 °C. Its IL-1β content was measured with a human IL-1β ELISA kit (BD OptEIA™; BD Pharmingen) according to the manufacturer’s instructions.

### 2.14. qRT-PCR

The A172, U373MG, and U87MG glioma cell lines were cultured in the presence of si-Tim-3 or si-Gal-9 for 3 d. On day 12, the glioma cells were lysed and their RNA was extracted with the RNeasy kit (Qiagen, Hilden, Germany) according to the manufacturer’s instructions. Extracted RNA yield, concentration, and purity were assayed with a Nanodrop spectrophotometer (Thermo Fisher Scientific). Two micrograms of high-quality RNA was used to synthesize cDNA with an iScript™ Select cDNA synthesis kit (Bio-Rad Laboratories, Hercules, CA, USA) and an oligo primer (Bio-Rad Laboratories). Quantitative PCR was then performed using a CFX96 Touch real-time PCR detection system (Bio-Rad Laboratories), HAVCR2-specific primers (Bio-Rad Laboratories), LGALS9-specific primers (Bio-Rad Laboratories), 100 ng of cDNA, and SYBR Green Supermix (Bio-Rad Laboratories). All PCR reactions were performed in triplicate. Data were analyzed by the 2^−ΔΔCt^ method. GAPDH (Bio-Rad Laboratories) was the reference gene.

### 2.15. Protein–Protein Interaction Analysis

A protein–protein interaction (PPI) analysis was conducted to localize physical connections between the Tim-3-Gal-9 axis and the NLRC4 inflammasomes. The PPI score was derived from the String database (https://string-db.org/, accessed on 21 November 2021). Its confidence score was ≤0.4 without text mining. A kinase-transcription factor (TF) axis analysis was conducted based on the PPI confidence score.

### 2.16. TF Prediction and Kinase-TF Axis Analysis

A TF prediction analysis was conducted to identify the TFs regulating the gene sets. A TF enrichment analysis was conducted in ChEA3 (https://maayanlab.cloud/chea3, accessed on 26 November 2021) using ENCODE ChIP-seq data and gene clusters 2. The kinases used in the kinase TF analysis were profiled in UniProt (https://www.uniprot.org/docs/pkinfam accessed on 22 November 2021). Based on the selected TFs and kinases, a network was constructed using iGraph v. 1.2.5 (https://igraph.org accessed on 20 November 2021). The PPI confidence score without text mining was >0.7.

### 2.17. Statistical Analysis

Significant differences between treatment means were determined by a paired *t*-test. One asterisk (*) denotes *p* < 0.05; two asterisks (**) denote *p* < 0.005; three asterisks (***) denote *p* < 0.0005.

## 3. Results

### 3.1. Tim-3/Gal-9 Are Significantly Correlated with the NLRC4 Inflammasome in Glioma

**Figure 1:**
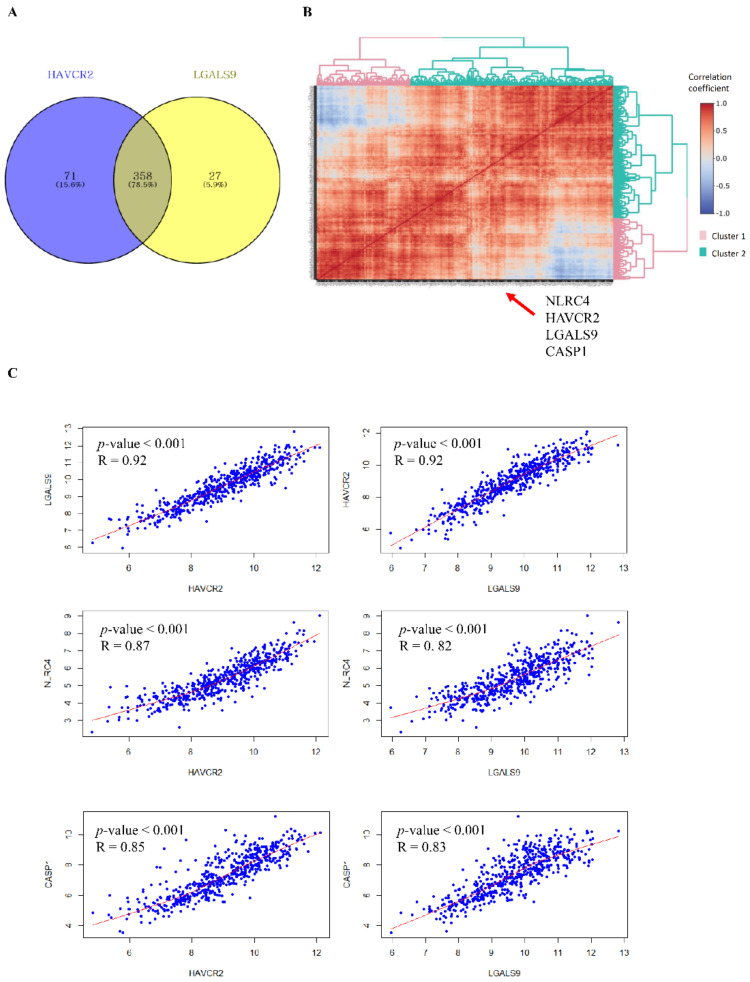
NLRC4 and CASP1 expression is highly positively correlated with those of Tim-3 and Gal-9. (**A**) There were 358 genes positively correlated with Tim-3 and Gal-9. (**B**) Correlation coefficient hierarchical clustering showing that NLRC4 inflammasome molecules are positively correlated with Tim-3 and Gal-9. (**C**) Pairwise dotplot of Tim-3, Gal-9, NLRC4, and CASP1. HAVCR2: Tim-3; LGALS9: Gal-9.

A correlation analysis was conducted to identify genes that are strongly positively associated with Tim-3 and Gal-9 (Table S4). We extracted genes with correlation coefficients >0.7. We found 429 and 385 genes positively correlated with Tim-3 and Gal-9, respectively (Figure 1A). Of these, 358 were positively correlated with both Tim-3 and Gal-9. Correlation coefficient hierarchical clustering was also performed to identify the genes most strongly positively correlated with Tim-3 and Gal-9. The selected genes were divided into two clusters. A pathway enrichment analysis was conducted on gene cluster 2 including Tim-3 and Gal-9. The significantly enriched biological process and cellular component were programmed cell death and the inflammasome complex, respectively (Supplementary Figure S1). Tim-3, Gal-9, NLRC4, and CASP1 were included in gene cluster 2 (Figure 1B). Gene and cluster information are presented in Table S5. The NLRC4 and CASP1 expression levels were strongly positively correlated with those of Tim-3 and Gal-9, respectively (Figure 1C).

### 3.2. Tim-3/Gal-9 and NLRC4 Inflammasome Expression Is Associated with Poor Survival in Patients with Glioma

**Figure 2:**
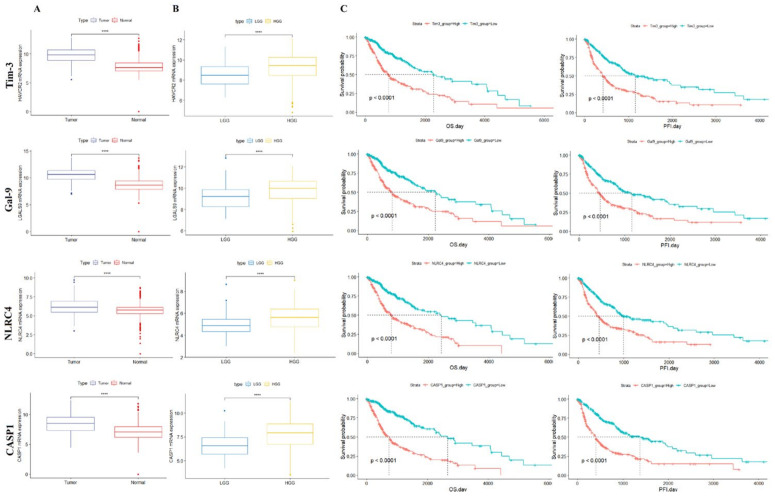
Survival analysis of Tim-3, Gal-9, NLRC4, and CASP1. (**A**) Comparison between tumor and normal tissue in terms of Tim-3, Gal-9, NLRC4, and CASP1 expression. (**B**) Comparison of gene expression levels in low-grade glioma (LGG) and high-grade glioma (HGG). (**C**) Survival analysis of high-expression and low-expression genes. OS: overall survival. PFI: progression-free interval.

As the correlations among Tim-3, Gal-9, NLRC4, and CASP1 were highly positive, we compared the expression levels of each gene in glioma and healthy tissue. The expression levels of all genes were significantly higher in tumor than normal tissue (Figure 2A). We also compared the expression levels of each gene between low-grade glioma (LGG; includes grade 2) and high-grade glioma (HGG; includes grades 3 and 4). The expression levels of all genes were higher in HGG than LGG (Figure 2B). In GBM, all four gene expressions were higher in the mesenchymal and neural type than the proneural type (Supplementary Figure S2A–D). We conducted analyses of the overall survival (OS) and progression-free interval (PFI) between two groups divided by their median gene expression levels. Downregulation of all four genes was associated with relatively better prognosis, OS, and PFI (Figure 2C).

### 3.3. Tim-3/Gal-9 and NLRC4 Inflammasome Are Upregulated in High-Grade Glioma Tissues

**Figure 3:**
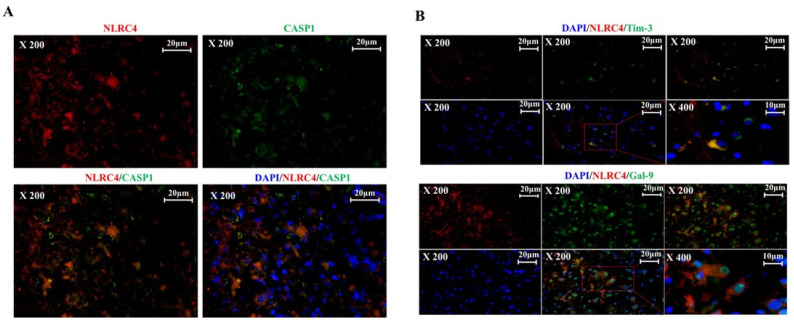

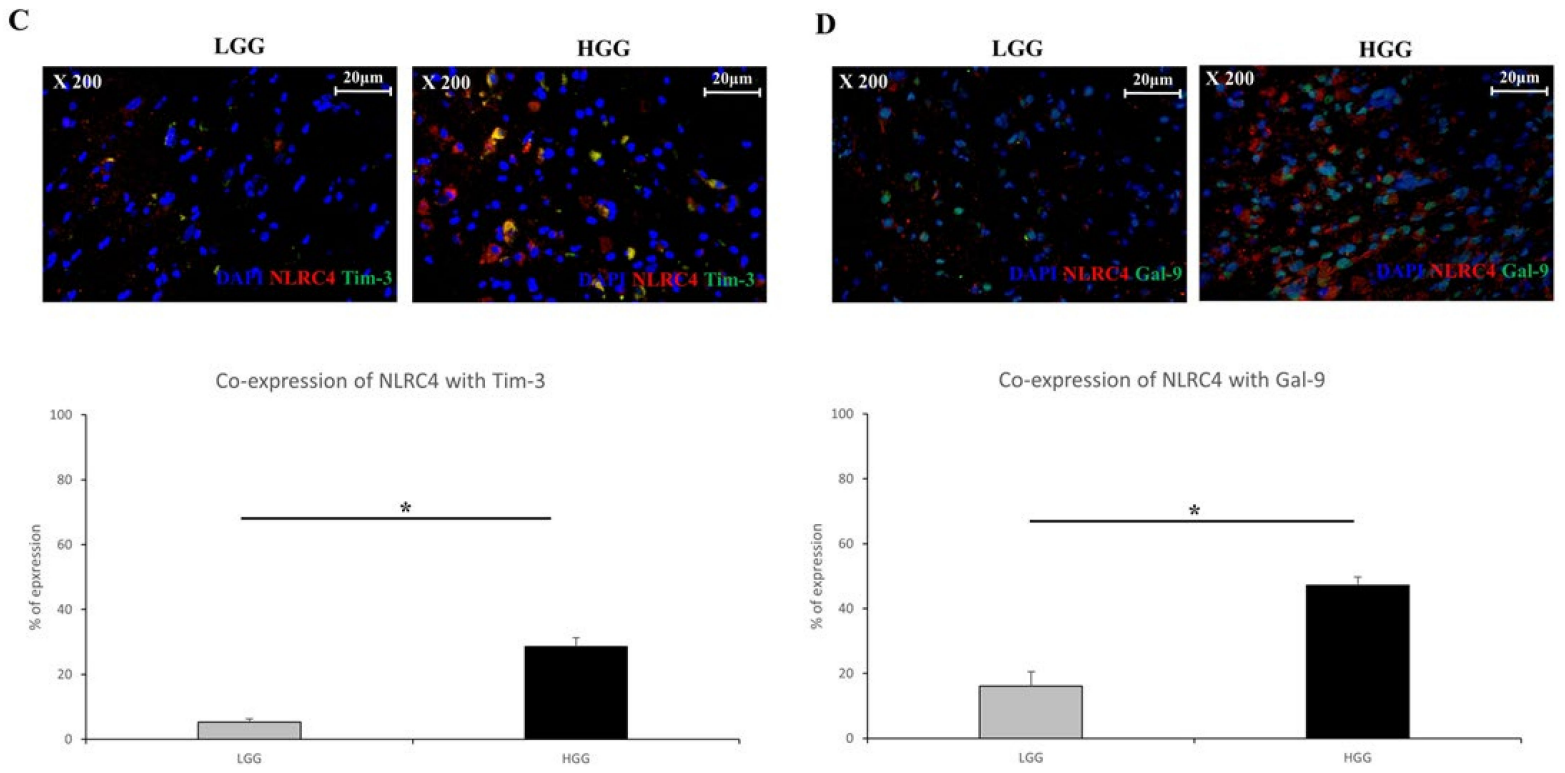
Tim-3/Gal-9 and NLRC4 inflammasome expression in glioma. (**A**,**B**) Human glioma specimens were analyzed by immunohistochemistry (IHC) to detect NLRC4 (red), caspase-1 (green), or Tim-3 (green). (**C**,**D**) NLRC4 co-expression with Tim-3 or Gal-9 for each grade (LGG: low-grade glioma; Grade 2/HGG: high-grade glioma; Grade 3 + Grade 4) was evaluated by IF and quantitated by ImageJ (National Institutes of Health, Bethesda, MD, USA). * *p* < 0.05; paired *t*-test.

An immunohistochemical examination was performed to establish whether the NLRC4 inflammasome is expressed in glioma tissues. The NLRC4 inflammasome co-localized with the NLRC4 and caspase-1 proteins. They were significantly co-expressed in glioma tissues (Figure 3A). Caspase-1 is crucial for inflammasome assembly and activity. We further questioned whether this caspase-1 is expressed in which cells in glioma. We performed co-staining of the caspase-1 with microglia (Iba1^+^) and astrocyte (GFAP^+^), which play important roles in the pathology of gliomas [28]. It co-localized with GFAP more than it did with Iba1. The NLRC4 inflammasome was detected mainly in astrocytes (Supplementary Figure S3A,B).

We previously confirmed through in silico analysis that Tim-3 and Gal-9 are significantly correlated with the NLRC4 inflammasome. Both co-expressed with NLRC4 protein in glioma tissues (Figure 3B). Gal-9 expression occurred on both the cell surface and the nucleus. Gal-9 was translocated into the nucleus [29] (Supplementary Figure S4A,B).

**Figure 4:**
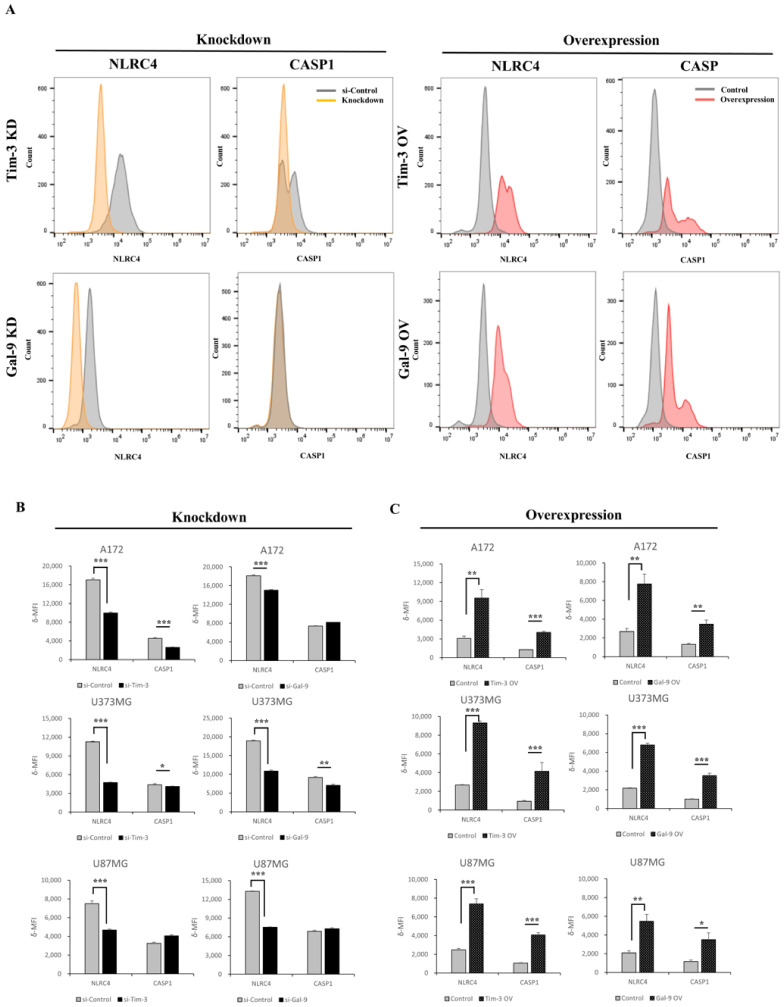
Tim-3/Gal-9 induce NLRC4 inflammasome in glioma cells. (**A**–**C**) Glioma cell lines were pretreated with Tim-3 and Gal-9 siRNA or pcDNA. On transfection day 2, A172, U373MG, and U87MG cells were collected and analyzed by flow cytometry. (**A**) Representative histogram of glioma cell line (A172) with NLRC4 and caspase-1 knockdown or overexpression. (**B**,**C**) NLRC4 and caspase-1 expression in glioma cells subjected to siRNA or pcDNA was calculated by flow cytometry (*n* = 3). * *p* < 0.05; ** *p* < 0.005; *** *p* < 0.0005; paired *t*-test.

We measured Tim-3, Gal-9, and NLRC4 expression at each stage of glioma. Between LGG and HGG, Tim-3/NLRC4 was upregulated from 5.34% ± 0.91% to 28.61% ± 2.63 and Gal-9/NLRC4 was upregulated from 16.13% ± 4.37% to 47.15% ± 2.54 (Figure 4C,D). Individual co-expression data for Figure 4C,D are shown in Supplementary Figure S5. The correlation between NLRP3 and Tim-3/Gal-9 was weaker than that between NLRC4 and Tim-3/Gal-9. Nevertheless, between HGG and LGG, NLRP3 co-expression with Tim-3 increased from 2.59% ± 0.52% to 38.13% ± 1.96, while its co-expression with Gal-9 increased from 34.56% ± 5.52% to 51.94% ± 1.37 (Supplementary Figure S6A–D). Hence, Tim-3/Gal-9 and NLRC4 inflammasome co-expression is correlated with glioma progression and occurs mainly in astrocytes.

### 3.4. Tim-3/Gal-9 Regulate the Expression of NLRC4 Inflammasome-Associated Molecules in Glioma

In silico and histological analyses confirmed that Tim-3/Gal-9 are significantly correlated with the NLRC4 inflammasome. We then explored whether Tim-3/Gal-9 regulate the expression of NLRC4 inflammasome-associated molecules. We used DepMap and a gene expression analysis based on Tim-3/Gal-9 expression to test this hypothesis in the glioma cell lines A172 and U87MG (Supplementary Figure S7). The test for U373MG was additionally conducted. We used flow cytometry to evaluate post-optimization and the validation of Tim-3/Gal-9 knockdown or overexpression and NLRC4 inflammasome expression in each glioma cell line (Supplementary Figure S8A,B). Tim-3 or Gal-9 downregulation significantly decreased NLRC4 and caspase-1 expression in glioma cells (Figure 4A,B). The NLRC4 inflammasome and caspase-1 expression levels were dramatically increased in glioma cells transfected with pcDNA harboring Tim-3 or Gal-9 (Figure 4C). We also examined the effects of Tim-3/Gal-9 on the NLRP3 inflammasome in glioma cells and showed that the NLRP3 inflammasome was regulated by Tim-3/Gal-9 similarly to the NLRC4 inflammasome (Supplementary Figure S9A,B).

### 3.5. Tim-3/Gal-9 Are Correlated with Caspase-1 Activation and Induces Programmed Cell Death in Glioma

**Figure 5:**
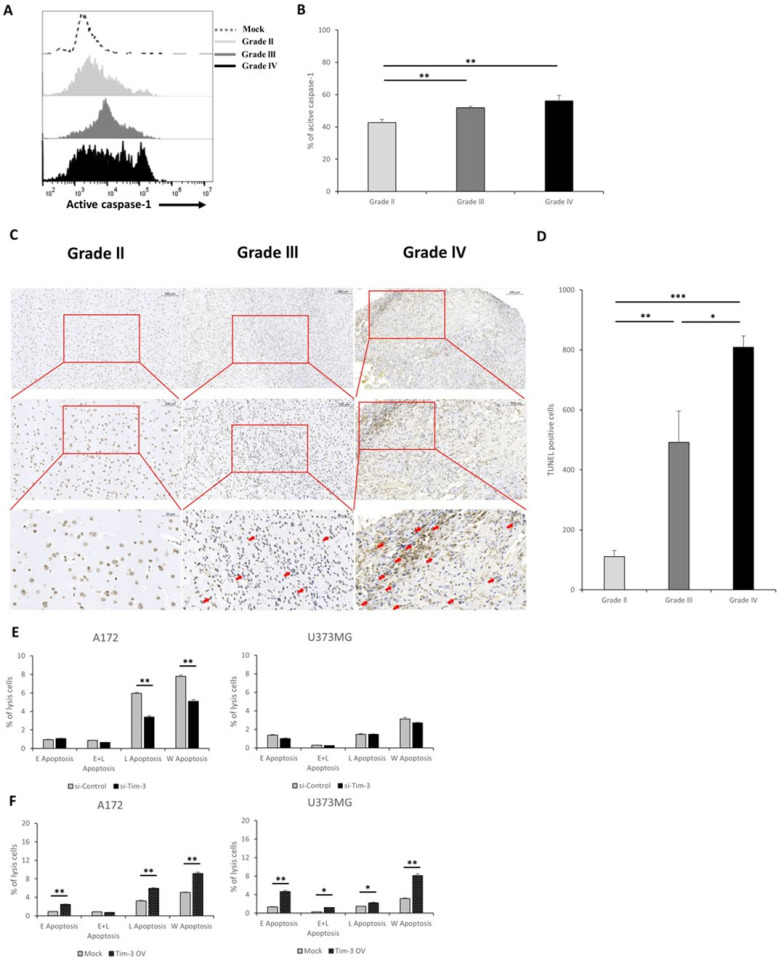
NLRC4 inflammasome activation and programmed cell death promoted by Tim-3/Gal-9. (**A**–**D**) Primary glioma cells including G2, G3, and G4 were stained with FLICA or TUNEL. (**A**) Representative histogram of active caspase-1 expression in each grade of primary glioma cells. (**B**) Quantitation of FLICA analysis was conducted by flow cytometry (*n* = 9). Histological analysis was performed by TUNEL staining to detect programmed cell death in glioma tissues. (**C**,**D**) Representative microscopy and quantitation of each glioma tissue grade. TUNEL-positive cells are highlighted by red arrows (blue color: DAPI/brown color: TUNEL) (**C**). (**E**,**F**) Glioma cell lines were pretreated with Tim-3 siRNA or pcDNA. On knockdown day 2, A172 and U373MG cells were collected and analyzed for programmed cell death by flow cytometry. Percentage cell death was assessed by flow cytometry after staining with Annexin V and 7-AAD (early apoptosis), Annexin V^+^7-AAD^−^ (late apoptosis), Annexin V^+^7-AAD^+^ (whole apoptosis); *n* = 3. * *p* < 0.05; ** *p* < 0.005; *** *p* < 0.0005; paired *t*-test.

The modulation of NLRC4 inflammasome expression by Tim-3/Gal-9 may also affect caspase-1 activity. The latter is a marker of inflammasome activation. A fluorescent-labeled inhibitor of caspase (FLICA) analysis of caspase-1 was performed on the primary cells of each glioma grade. Glioma malignancy and caspase-1 activity were positively correlated (Figure 5A,B). The activity of the NLRC4 inflammasome, including the activity of caspase-1, is closely related to the inflammatory microenvironment [30]. We further confirmed through H&E staining whether an inflammatory microenvironment was revealed in the glioma tissue. As a result, the higher the grade of the glioma, the stronger the characteristics such as angiogenesis and mitosis, which are characteristics of the inflammatory microenvironment (Supplementary Figure S10).

In general, when caspase-1, an indicator of NLRC4 inflammasome activation, is activated, secretion of proinflammatory cytokines including IL-1β and IL-18 increases [31]. Thus, active caspase-1 in the NLRC4 inflammasome may induce the secretion of the proinflammatory cytokines IL-1β and IL-18. Despite active caspase-1 upregulation (Figure 5A,B), there were no significant differences in IL-1β secretion in response to Tim-3 or Gal-9 knockdown in any of the glioma cell lines (Supplementary Figure S11A,B).

We investigated the effects of Tim-3/Gal-9 on programmed cell death in glioma. This process indicates NLRC4 inflammasome activation. An in situ cell death analysis showed that the number of cells dying in the human glioma tissues increased with glioma malignancy (Figure 5C,D). Both downregulated and upregulated Tim-3 and Gal-9 regulated glioma cell survival (Figure 5E,F). Thus, Tim-3 and Gal-9 may be significantly correlated with caspase-1 activation and, by extension, programmed cell death in glioma.

### 3.6. Tim-3/Gal-9-NLRC4 Inflammasome Network Landscape Determined by PPI

**Figure 6:**
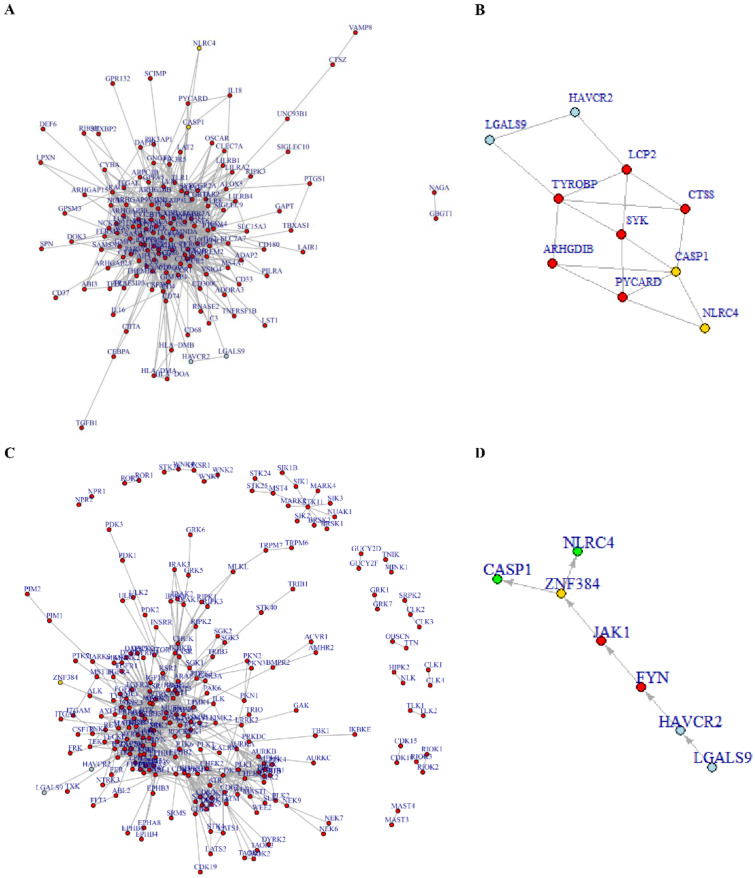
Tim-3/Gal-9 are directly and indirectly connected with the NLRC4 inflammasome. Protein–protein interaction (PPI)-based network analysis using gene cluster 2 showed that Tim-3/Gal-9 are directly connected with the NLRC4 inflammasome complex (**A**). Reconstructed network using molecules included in shortest path. Blue: Tim-3/Gal-9. Red: mediator molecules. Yellow: NLRC4 inflammasome molecules (**B**). Transcription factor (TF) prediction analysis by CHEA3 was performed using gene cluster 2. ZNF384 was the only TF regulating NLRC4 and CASP1 expression (**C**). Tim-3/Gal-9-kinase-TF axis analysis was conducted along with PPI to investigate the regulation of NLRC4 inflammasome complex molecule expression. The Tim-3/Gal-9-LCK-JAK1-ZNF384 axis was the only pathway regulating NLRC4 inflammasome expression. Blue: Tim-3/Gal-9. Red: kinase. Yellow: TF. Green: NLRC4 inflammasome molecules (**D**).

We demonstrated that Tim-3/Gal-9 positively regulate NLRC4 inflammasome expression and activity. Hence, we also explored whether there were direct and/or indirect connections between Tim-3/Gal-9 and the NLRC4 inflammasome. We performed a protein–protein interaction (PPI)-based network analysis using gene cluster 2 to infer any direct connections between Tim-3/Gal-9 and NLRC4 inflammasome activation. NLRC4 inflammasome-associated molecules were physically connected to Tim-3/Gal-9 but they did not directly interact (Figure 6A). The molecules included in the network between Tim-3 or Gal-9 and NLRC4 or CASP1 were extracted using the shortest path’s function and network reconstruction (Figure 6B). We analyzed the regulation of NLRC4 and CASP1 expression using the Tim-3/Gal-9-kinase-TF axis by bioinformatical analysis. Sixteen TFs were significantly enriched with gene cluster 2. Only ZNF384 regulated both NLRC4 and CASP1. A pathway map was plotted based on the PPI-based network analysis and included Tim-3/Gal-9, kinases, and ZNF384 (Figure 6C). The starting point was Tim-3/Gal-9 and the end point was ZNF384. A single pathway was inferred and it consisted of Tim-3/Gal-9-FYN-JAK1-ZNF384 (Figure 6D). Therefore, we predicted that NLRC4 inflammasome molecules are positively regulated by the foregoing pathway.

## 4. Discussion

Previous studies have suggested that Tim-3/Gal-9 may affect the inflammatory microenvironment in different tumors [8,16]. However, it is unknown whether they have similar influences in glioma. In the present study, we showed that Tim-3/Gal-9 are significantly correlated with the NLRC4 inflammasome. Tim-3/Gal-9 promote NLRC4 inflammasome formation and activation and trigger inflammation.

Recent studies have reported on the association between Tim-3/Gal-9 expression and cancer prognosis. Tim-3 upregulation was related to poor OS, local lymph node invasion, tumor grade, and PD-1 expression in gastric cancer [32]. It was also associated with poor prognosis in colon cancer [33]. Another study showed that blocking Tim-3 induced tumor volume regression in murine glioma [34]. Tim-3 and Gal-9 expression levels were higher in HGG than LGG [34]. Our analysis of TCGA GBMLGG data demonstrated that Tim-3/Gal-9 expression increased with tumor grade. We performed a correlation analysis to identify the genes associated with Tim-3/Gal-9 expression. A GO analysis using genes positively correlated with Tim-3/Gal-9 revealed programmed cell death under the biological process and the inflammasome complex under the cellular component. The inflammasome complex included NLRC4, CASP1, and PYCARD (Supplementary Figure S1). NLRC4 and CASP1 expression increased with histological grade. Based on the in silico results, we conducted an experimental validation and found that Tim-3/Gal-9 and NLRC4 were significantly positively correlated with each other, as well as glioma malignancy. These results suggest that Tim-3/Gal-9 are closely linked with the NLRC4 inflammasome in glioma.

By contrast, Tim-3/Gal-9 and the NLRC4 inflammasome were expressed mainly in the astrocytes. Hence, these cells play a negative role in the glioma TME [35]. Gal-9 was localized to the cell membrane and the nucleus (Supplementary Figure S5) and might be translocated to the nucleus by interacting with NF-IL6 [29]. However, this putative mechanism requires empirical verification. Tim-3 and Gal-9 knockdown with siRNA disclosed that they were expressed on the membranes and in the cytoplasms of glioma cells (data not shown). The intracellular Tim-3 and Gal-9 protein expression levels were lower than those on the membranes. Nevertheless, this pattern was not uniform among all cells. These findings suggest that intracellular Tim-3/Gal-9 may indirectly induce the inflammasome.

We confirmed that caspase-1 activity and programmed cell death downstream of the NLRC4 inflammasome increased with tumor malignancy because of Tim-3/Gal-9. Although we did not specifically confirm what type of cells apoptosis occurred in the glioma tissues according to each grade, programmed cell death proceeded in glioma cells with knockdown Tim-3 in vitro. From the viewpoint of the tumor microenvironment, an apoptosis signal in the tumor cells can further accelerate inflammation and tumor progression via interaction with neighboring cells, which, in turn, can benefit tumor growth [36,37,38]. Meanwhile, proinflammatory cytokine secretion occurs in response to caspase-1 activation and NLRC4 inflammasome oligomerization [39]. However, the secretion of proinflammatory cytokines such as IL-1β did not significantly differ among treatments. Earlier studies on inflammasome stimulation generally used LPS or bacteria [40]. Nevertheless, we did not apply either of these to elucidate the glioma TME window or the effects of Tim-3/Gal-9 on the NLRC4 inflammasome. We propose that a strong signal-like microbial stimulus mediated by the Toll-like receptor (TLR) family is required for inflammatory protein secretion (Figure 5).

Our experiments showed that Tim-3/Gal-9 are connected with NLRC4 inflammasome expression and activation. Previous studies have clarified precise mechanisms for the interactions between Tim-3/Gal-9 and the NLRC4 inflammasome in immune cells but not in tumors [41,42]. For these reasons, we conducted a PPI-based mechanistic study on the associations between Tim-3/Gal-9 and NLRC4 inflammasome activation and expression with a computational method. The PPI network analysis used gene cluster 2 and revealed that NLRC4 inflammasome molecules and Tim-3/Gal-9 are physically linked with LCP2, ARHGDIB, and TYROBP. Thus, the foregoing molecules can directly activate CASP1. However, Tim-3 binds Gal-9, which interacts with the kinase FYN [43]. The NLRC4 and CASP1 expression levels were modulated by ZNF384. The latter was identified by TF enrichment analysis with gene cluster 2. A PPI analysis between FYN and JAK1 predicted that the latter can be a bridge molecule. Tim-3/Gal-9 can positively regulate NLRC4 and CASP1 via the FYN-JAK1-ZNF384 pathways. Although our results were from computational inference analysis, we believe that Tim-3/Gal-9 can positively regulate NLRC4 and CASP1 via the FYN-JAK1-ZNF384 pathway, and validation with further experiments are needed.

## 5. Conclusions

This study demonstrated that Tim-3/Gal-9 are correlated with the glioma NLRC4 inflammasome. They promote the latter by modulating NLRC4 or caspase-1 expression and activation, which lead to programmed cell death. The influences of Tim-3/Gal-9 on the NLRC4 inflammasome may be used in glioma diagnosis and treatment targeting Tim-3/Gal-9 or the chronic inflammatory microenvironment. An inferential PPI analysis between Tim-3/Gal-9 and the NLRC4 inflammasome predicted that Tim-3/Gal-9 could trigger NLRC4 inflammasome activation. Clarification of the connection between the immune checkpoint and the inflammatory response indicates a novel direction for glioma therapy, could lead to the discovery of other target molecules and anticancer mechanisms, and may be applied against other types of cancer.

## Data Availability

Not applicable.

## Supplementary Materials

The following supporting information can be downloaded at: https://www.mdpi.com/article/10.3390/ijms23042028/s1.
