# Supplementary material for: Association of Tim-3/Gal-9 Axis with NLRC4 Inflammasome in Glioma Malignancy: Tim-3/Gal-9 Induce the NLRC4 Inflammasome
Source: Int J Mol Sci. 2022 Feb 12;23(4):2028. doi: 10.3390/ijms23042028 (PMC8878774; doi:10.3390/ijms23042028)
Supplement: Supplementary file 1 [file ijms-23-02028-s001.zip › ijms-1554252-Supplementary.pdf]

Supplementary

Association of Tim-3/Gal-9 axis with NLRC4 Inflammasome in Glioma Malignancy: Tim-3/Gal-9 Induces the NLRC4 Inflammasome

Jeong Min Sim[1,2,†], JeongMan Park[1,2,†], Suwan Kim[1], Sojung Hwang[1,3], Kyoung Su Sung[4], Jung-Eun Lee[5], Seung Ho Yang[5], Kyunggi Cho[1], Sung Hwan Lee[6], Jong-Seok Moon[7], Ju Won Ahn[1,*], Jaejoon Lim[1,2,*]

1 Department of Neurosurgery, Bundang CHA Medical Center, CHA University, Seongnam, Republic of Korea.
2 Department of Biomedical Science, College of Life Science, CHA University, Seongnam, Republic of Korea.
3 Global Research Supporting Center, Bundang CHA Medical Center, CHA University College of Medicine, Seongnam, Republic of Korea
4 Department of Neurosurgery, Dong-A University Hospital, Dong-A University College of Medicine, Busan, Republic of Korea
5 Department of Neurosurgery, St. Vincent's Hospital, College of Medicine, The Catholic University of Korea, Seoul, Republic of Korea
6 Department of Surgery, Bundang CHA Medical Center, CHA University, Seongnam, Republic of Korea.
7 Soonchunhyang Institution of Medi-bio Science (SIMS), Soonchunhyang University, Cheonan-si, Chungcheongnam-do, Republic of Korea

†Contributed equally as co-first authors.

*Contributed equally as co-last authors.

***Corresponding authors**:

Jaejoon, Lim, M.D., Ph.D. Department of Neurosurgery, Bundang CHA Medical Center, CHA University College of Medicine, Yatap-dong 59, Seongnam, 13496, Republic of Korea. Tel.: +82-31-780-5688; Fax: +82-31-780-5269. E-mail: coolppeng@chamc.co.kr

Ju Won Ah, Ph.D. Department of Neurosurgery, Bundang CHA Medical Center, CHA University College of Medicine, Yatap-dong 59, Seongnam, 13496, Republic of Korea. Tel.: +82-31-780-5688; Fax: +82-31-780-5269. E-mail: eugene@chauniv.ac.kr

Supplementary method.

Hematoxylin and eosin (H&E) staining

Human glioma brain tissues (n=9) were harvested and fixed in 4% formaldehyde. After preparation for paraffin embedded glioma block, glioma blocks were serially sectioned at 4 μm. To perform H&E histological study, The sections were stained with H&E. The sections were examined under a confocal laser scanning microscope (Zeiss LSM; Carl Zeiss AG, Jena, Germany)

Table S1. Clinical information of glioma patients

Patient	Diagnosis	Grade	Age	Sex	IDH mutaton	Ki-67	1p19q co-deletion	MGMT methylation	Overall survival (month)
P1	Diffuse astrocytoma	2	27	F	wild-type	2%	negative	negative	94+
P2	Diffuse astrocytoma	2	34	M	wild-type	5%	negative	negative	85+
P3	Diffuse astrocytoma	2	42	M	wild-type	4%	negative	negative	40
P4	Anaplastic astrocytoma	3	36	M	wild-type	10%	negative	positive	25
P5	Anaplastic astrocytoma	3	52	M	wild-type	7%	negative	negative	62+
P6	Anaplastic astrocytoma	3	63	F	wild-type	8%	negative	positive	31
P7	Glioblastoma	4	27	M	wild-type	30%	negative	positive	16
P8	Glioblastoma	4	63	M	wild-type	30%	negative	positive	9
P9	Glioblastoma	4	39	F	wild-type	20%	negative	negative	32

Table S2. Tim-3 and Gal-9 siRNA sequence and pcDNA primer information.

Type	Gene	Direction	Sequence
siRNA	Tim-3	Duplex	5′ GAAAUUCGGGCACAUCAUA 3′
			5′ UAUGAUGUGCCCGAAUUUC 3′
	Gal-9	Duplex	5′ CAUUGAAUAACAGCCAAGU 3′
			5′ ACUUGGCUGUUAUUCAAUG 3′
pcDNA	Tim-3	F′	5′ CAGGTGTCCACTCCCAGGTCCAAG 3′
		R′	5′ GGCAACTAGAAGGCACAGTCGAGG 3′
	Gal-9	F′	5′ CAGGTGTCCACTCCCAGGTCCAAG 3′
		R′	5′ GGCAACTAGAAGGCACAGTCGAGG 3′

Table S3. Antibody information

Isotype control	Fluorescence	Antibody isotype	Source
Goat IgG	Unconjugation	Goat IgG	Invitrogen (Carlsbad, CA)
Mouse IgG	Unconjugation	Mouse IgG	Invitrogen
Mouse IgG	PerCP710	Mouse IgG	Invitrogen
Mouse IgG	Alexa680	Mouse IgG	Invitrogen
Mouse IgG	PerCP	Mouse IgG	Invitrogen
Rat IgG	FITC	Rat IgG	BD Bioscience (San Jose, CA)
Rat IgG	PE	Rat IgG	Abcam (Cambridge, UK)
Rabbit IgG	FITC	Rabbit IgG1	BD Bioscience
**Primary antibody**	**Fluorescence**	**Antibody isotype**	**Source**
Tim-3	PE	Rat IgG	R&D System (Minneapolis, MN)
Gal-9	Unconjugation	Polyclonal Goat IgG	R&D System
NLRC4	FITC	Rabbit IgG	Novus Biologicals (Centennial, CO)
NLRP3	FITC	Rat IgG	R&D System
Caspase-1	Unconjugation	Mouse IgG1	Genetex (Irvine, CA)
Iba1	Alexa680	Mouse IgG	Santa Cruz Biotechnology (Dallas, TX)
GFAP	PerCP	Mouse IgG	Novus Biologicals
**Secondary antibody**	**Fluorescence**	**Antibody isotype**	**Source**
Rat anti-Mouse IgG	PerCP 710	Rat IgG1	BD Bioscience
Rabbit anti-Goat IgG	APC	Rabbit IgG	Cayman Chemical (Ann Arbor, MI)

Table S4. Correlation analysis of Tim-3 and Gal-9. HAVCR2: Tim-3. LGALS9: Gal-9. *p*: p-value. (Excel file)

Table S5. Correlation coefficient hierarchical clustering. (Excel file)

Category	Term	*p*	-LOG10(*p*)
CC	Inflammasome complex	0.0013518	2.8691028
	NLRP1 inflammasome complex	0.0016135	2.7922379
	AIM2 inflammasome complex	0.0016135	2.7922379
	IPAF inflammasome complex	0.0025182	2.5989061
	NLRP3 inflammasome complex	0.0079497	2.0996471
BP	Positive regulation of cell death	6.87E-06	5.1633026
	Regulation of cell death	4.37E-05	4.3593815
	Regulation of programmed cell death	5.20E-05	4.2843452
	Programmed cell death	6.54E-05	4.1847178
	Regulation of apoptotic process	9.98E-05	4.0008702
	Apoptotic process	3.65E-05	4.4379942
	Positive regulation of apoptotic process	8.44E-05	4.0738776
	Regulation of apoptotic process	9.98E-05	4.0008702

Supplementary Figure S1. Pathway enrichment analysis using GO biological process and cellular components. CC: cellular components; BP: biological process; *p*: p-value.

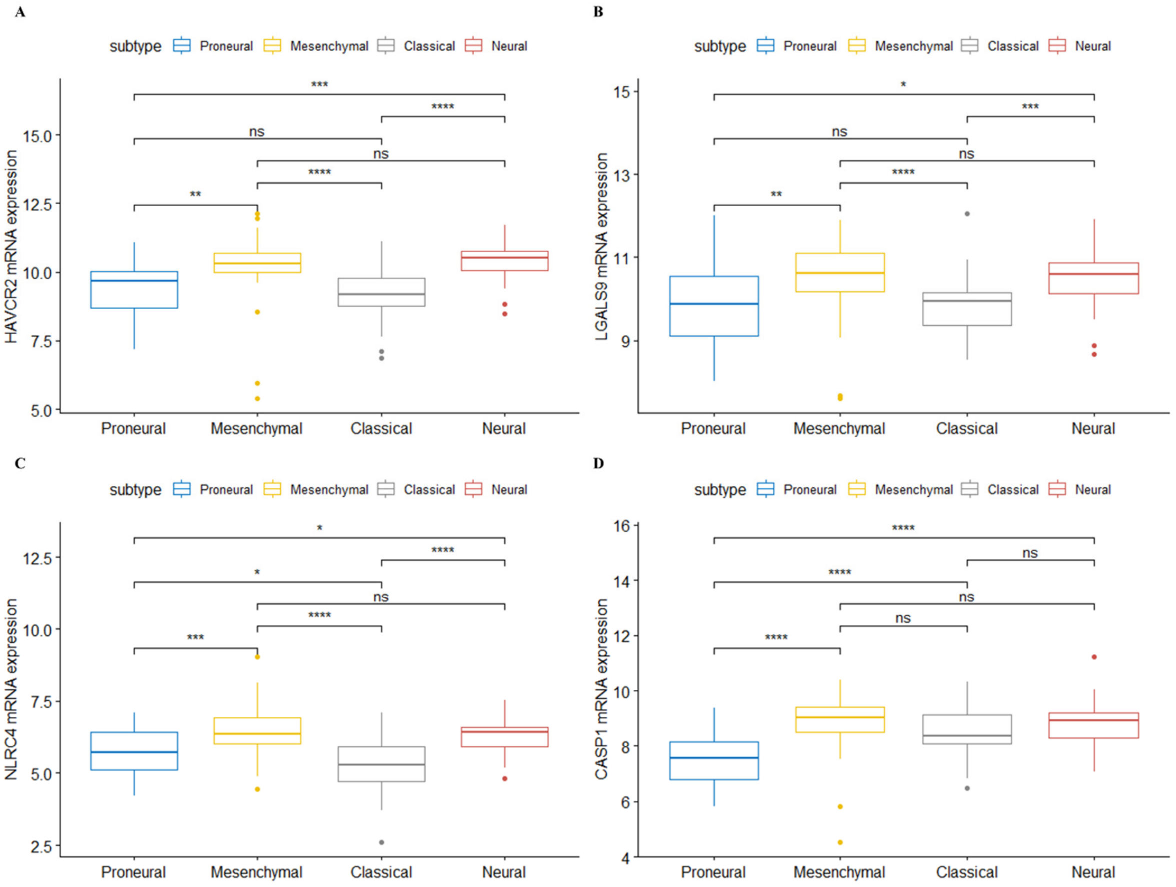

Supplementary Figure S2. The expression of Tim-3, Gal-9, NLRC4, and CASP1 in four subtypes of GBM. (A-D) Comparing the expression of Tim-3, Gal-9, NLRC4, and CASP1 between subtypes of GBM.

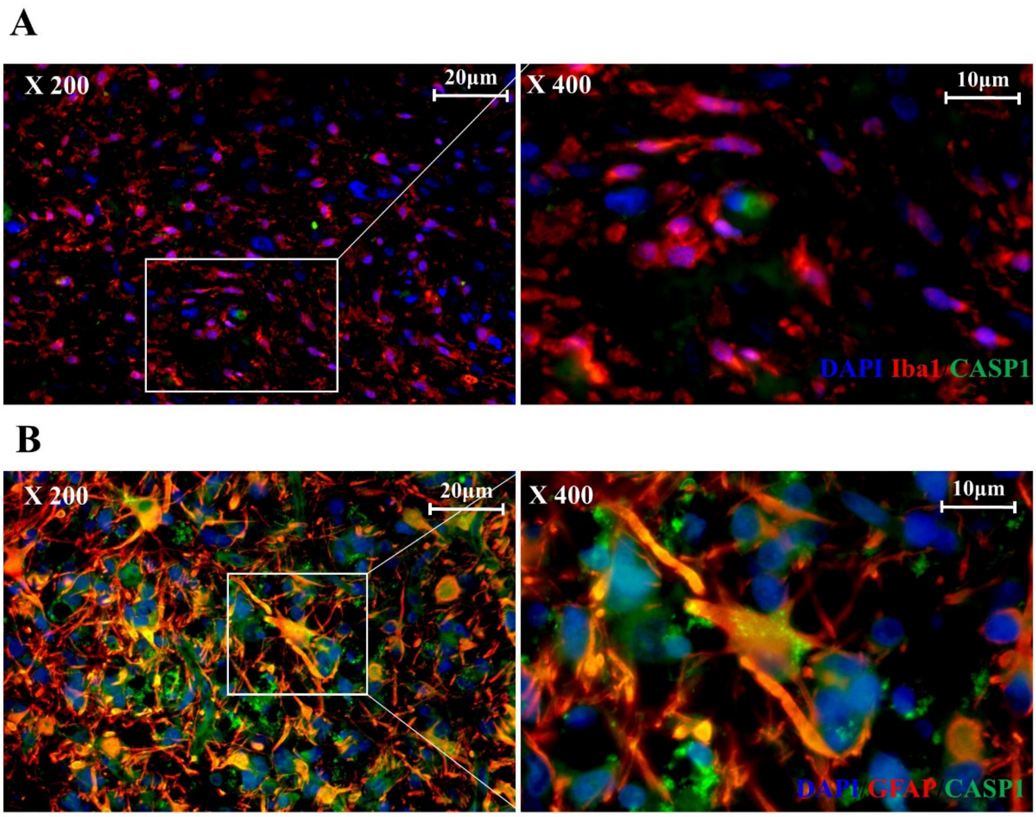

Supplementary Figure S3. NLRC4 inflammasome is expressed mainly in astrocytes in glioma TME. (A–B) To identify cells in which NLRC4 inflammasome is expressed, human glioma specimens were analyzed by immunohistochemistry (IHC) for caspase-1 (green), Iba1 (red), or GFAP (red). Grades 2, 3, and 4 glioma tissues were analyzed in triplicate. Representative image is G4 glioma.

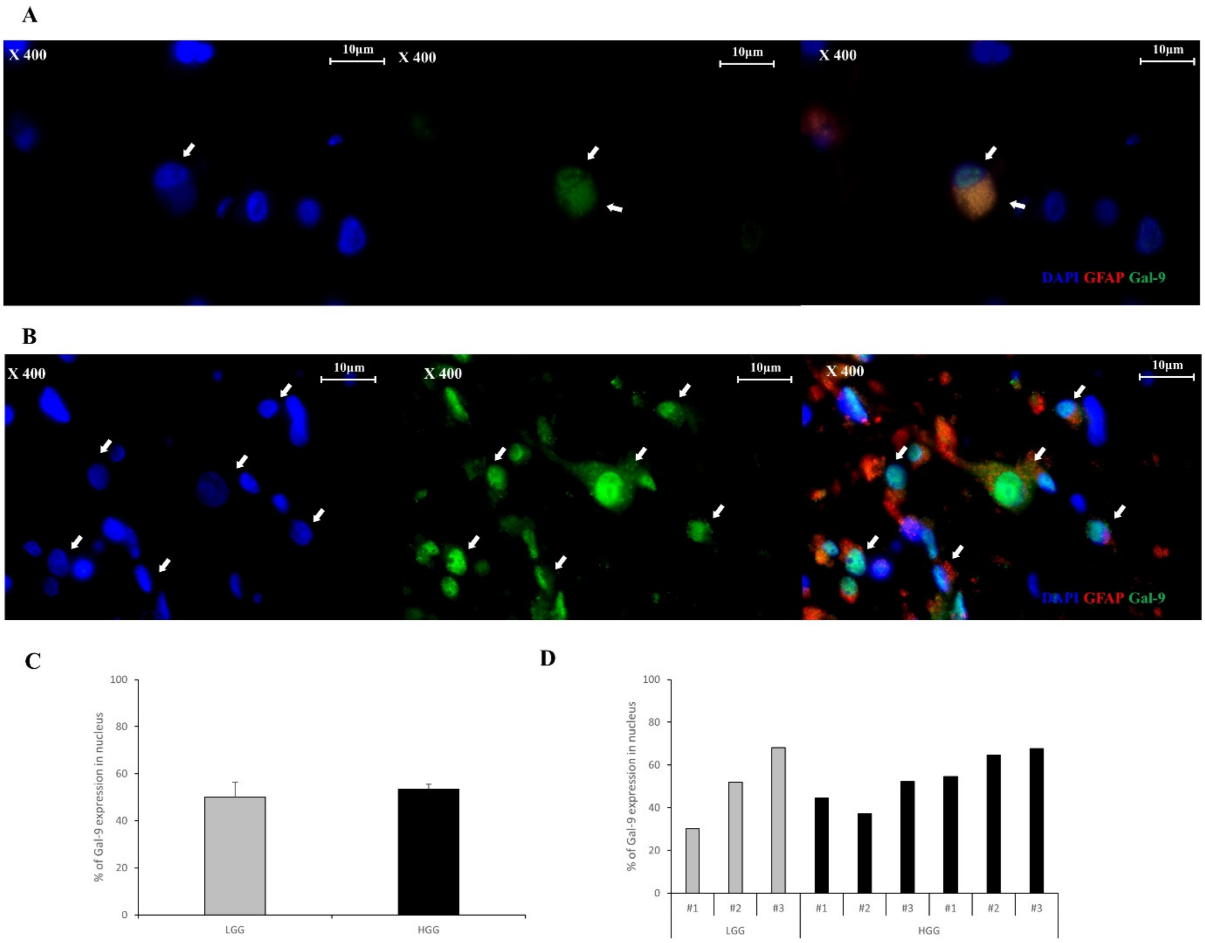

Supplementary Figure S4. Gal-9 is translocated to glioma cell nuclei. (A–D) Glioma specimens were stained with Gal-9 (green) or GFAP (red). Graphical images of LGG (A) and HGG (B) are shown and highlighted with white arrow indicating Gal-9 translocation to nucleus. (C) Quantitation of Gal-9 translocated to glioma cell nuclei were performed with ImageJ (n = 9). (D) Individual data are shown.

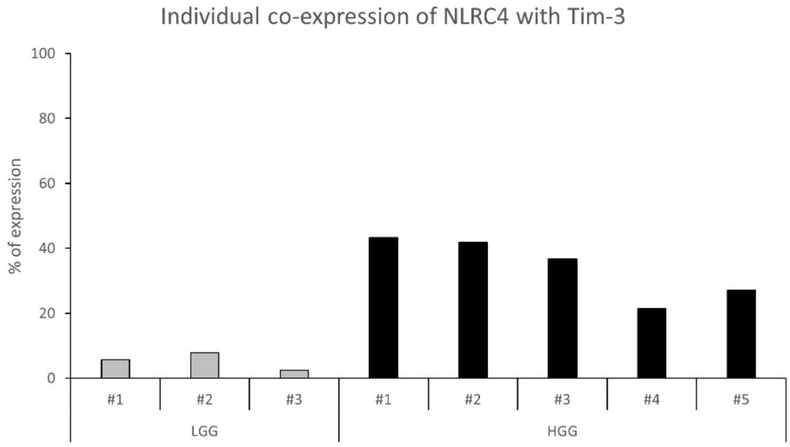

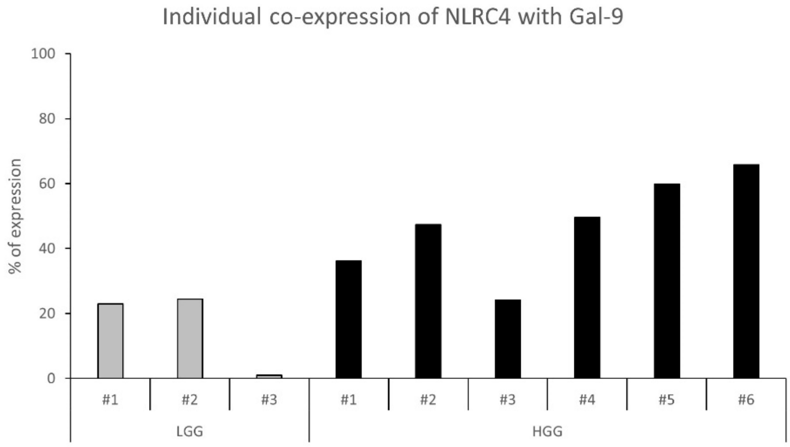

Supplementary Figure S5. Individual results for Tim-3/Gal-9 and NLRC4 inflammasome expression in glioma tissue. Human glioma specimens were analyzed by immunohistochemistry (IHC) to detect NLRC4 (red), Gal-9 (green) or Tim-3 (green). NLRC4 co-expression with Tim-3 or Gal-9 for each grade (LGG: low-grade glioma; Grade 2/HGG: high-grade glioma; Grade 3 + Grade 4) was evaluated by IF and quantitated by ImageJ (National Institutes of Health, Bethesda, MD, USA). Individual co-expression is shown.

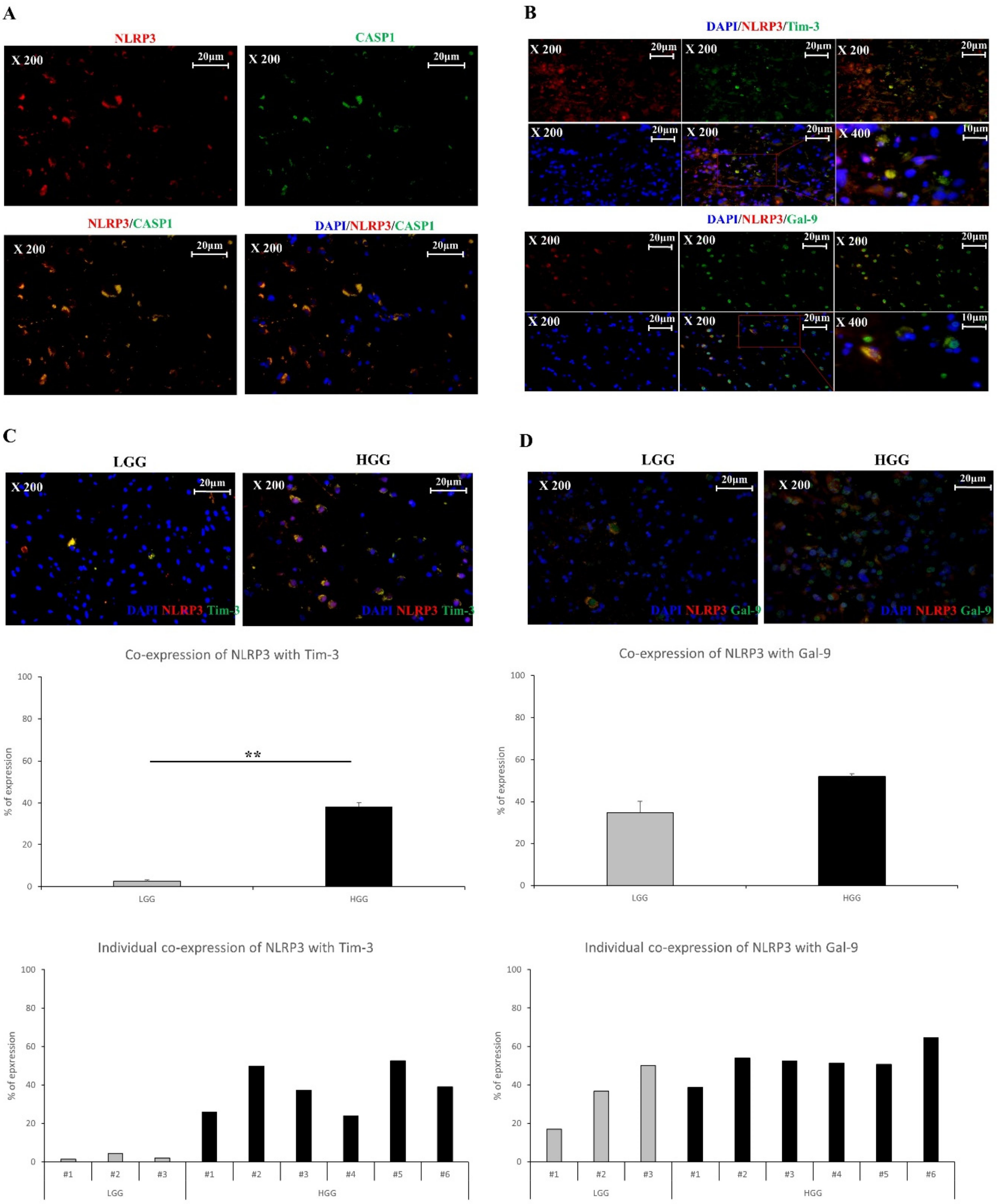

Supplementary Figure S6. Tim-3/Gal-9 and NLRP3 inflammasome co-expression. (A–B) Human glioma tissues (n=9) were analyzed by immunohistochemistry (IHC) for NLRP3 (red), caspase-1 (green), or Tim-3 (green). (C–D) NLRP3 co-expression with Tim-3 or with Gal-9 at each glioma grade was assessed by IHC and quantitated by ImageJ. Individual co-expression is shown at bottom. **$P < 0.005$; paired *t*-test.

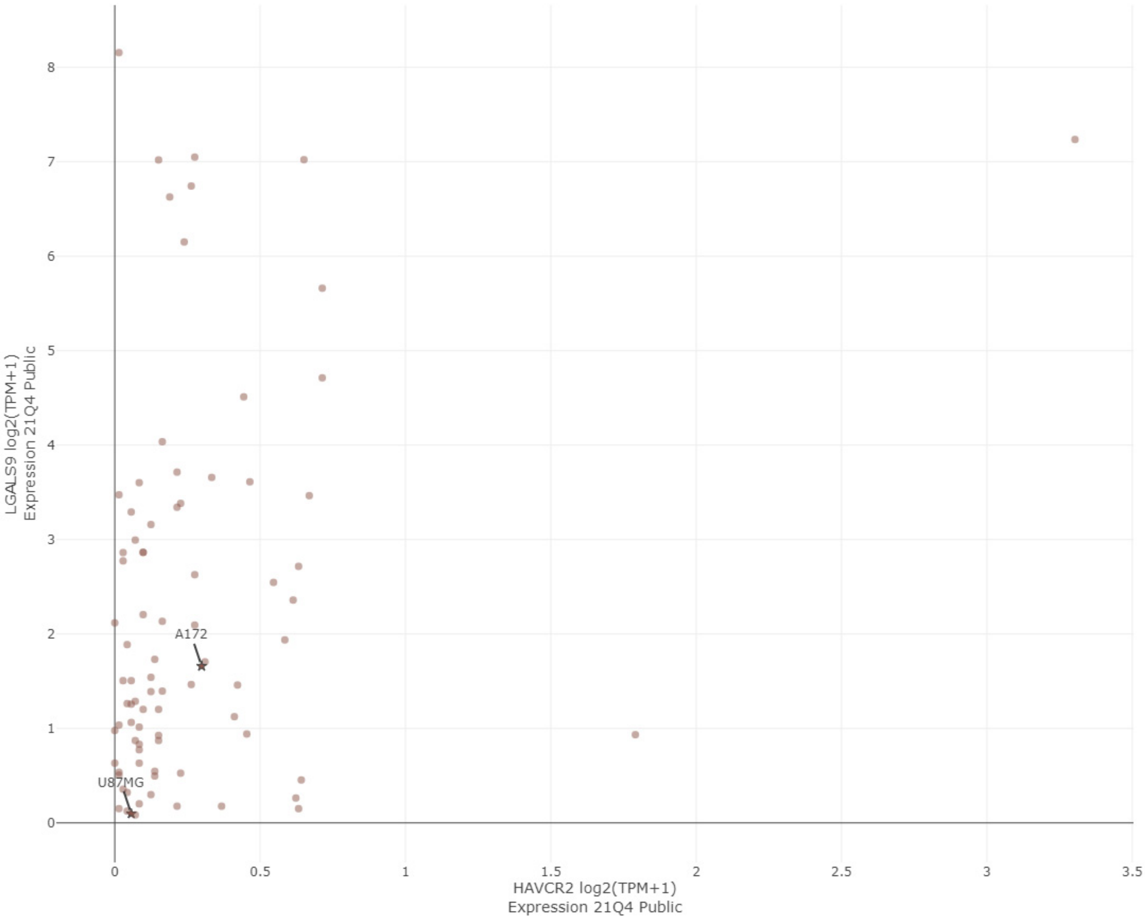

Supplementary Figure S7. Expression analysis of Tim-3/Gal-9. The mRNA expression of Tim-3/Gal-9 in A172, and U87MG cell lines with DepMap.

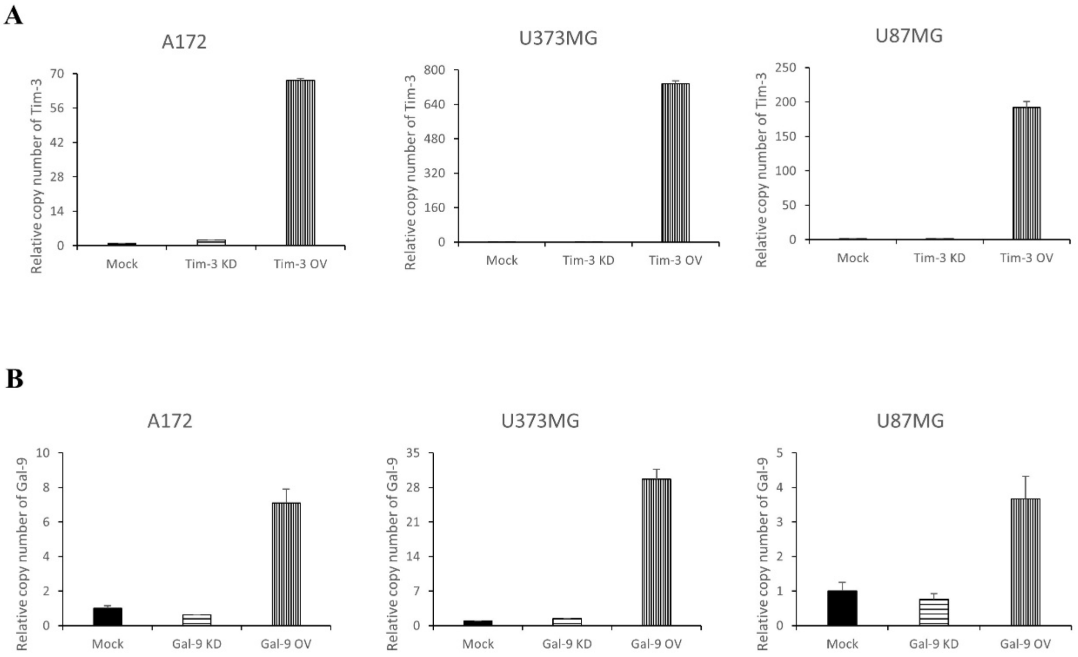

Supplementary Figure S8. Validation of Tim-3/Gal-9 knockdown or overexpression. (A–B) Glioma cell lines were treated with Tim-3 and Gal-9 siRNA or pcDNA and cultivated for 2 d. On day 2, total RNA was isolated and Tim-3 or Gal-9 expression was evaluated by qRT-PCR. Expression was normalized to that of GAPDH and quantitated by the $2^{-\Delta\Delta Ct}$ method. Data are derived from triplicate experiments (n = 3).

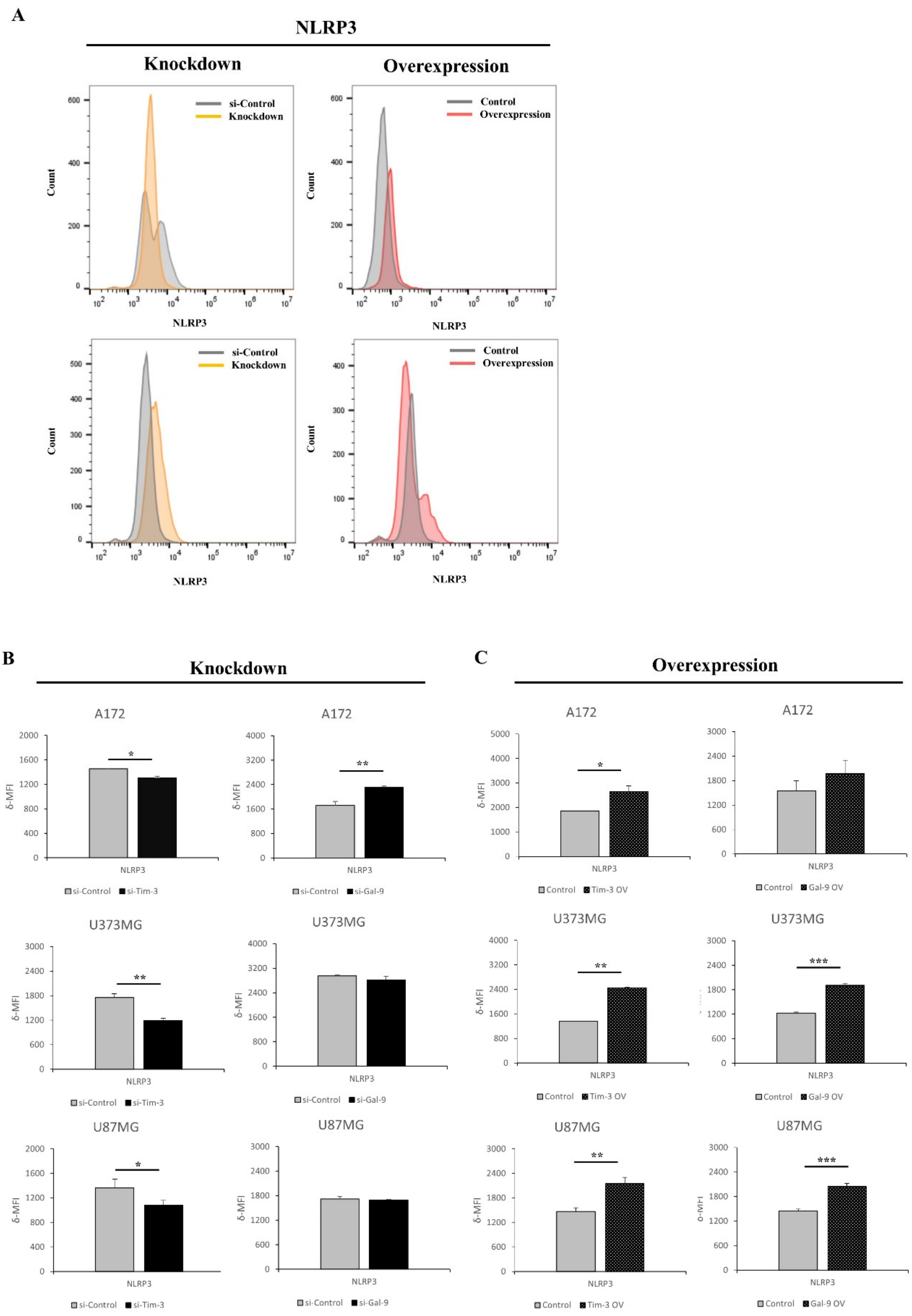
A
NLRP3
Knockdown
Overexpression
si-Control
Knockdown
Control
Overexpression
Count
NLRP3
B
Knockdown
C
Overexpression
A172
U373MG
U87MG
δ-MFI
NLRP3
si-Control
si-Tim-3
si-Gal-9
Control
Tim-3 OV
Gal-9 OV
*
**
***

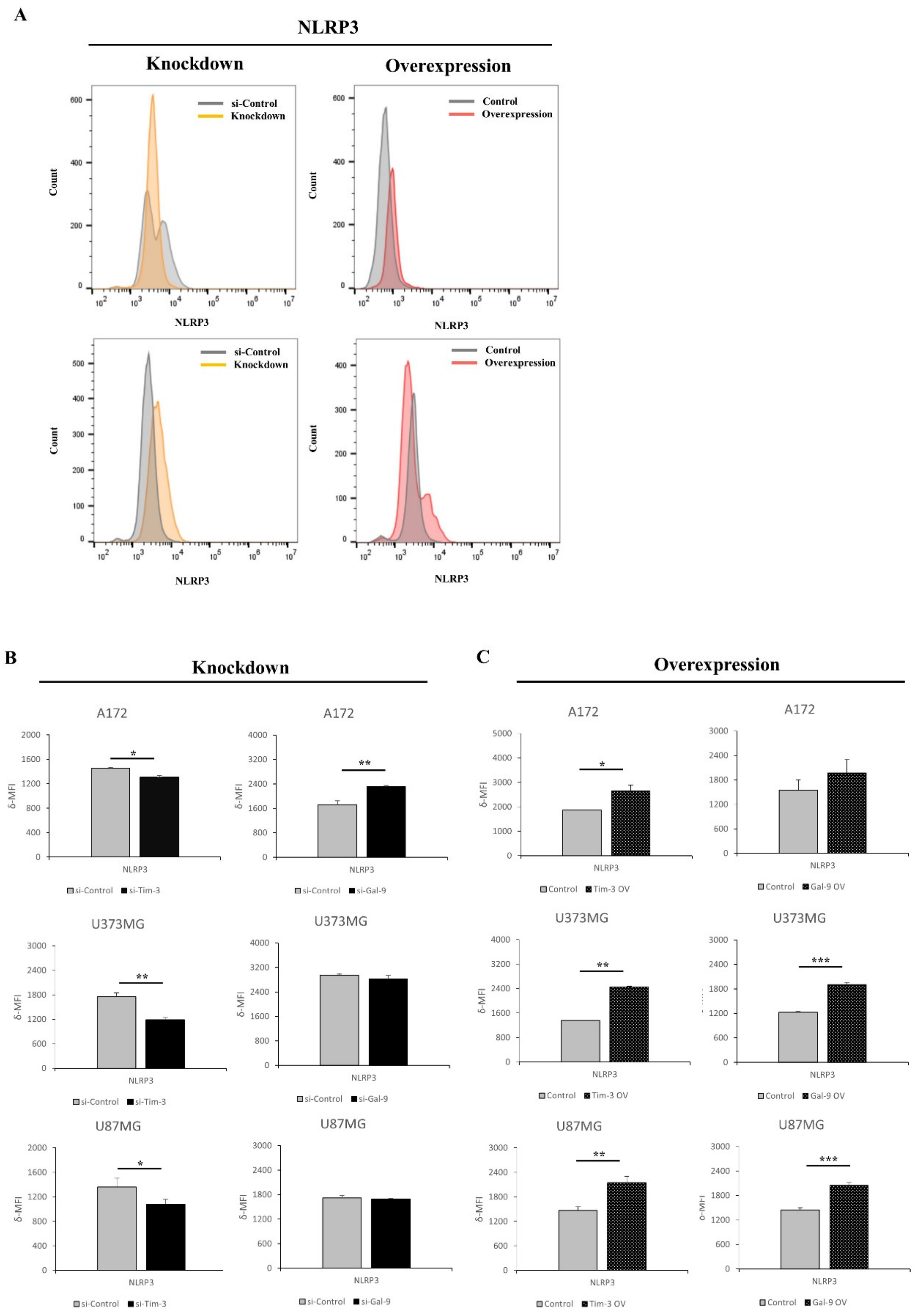

Supplementary Figure S9. Tim-3/Gal-9 regulates NLRP3 inflammasome expression. (A–C) Glioma cell lines were pretreated with Tim-3 and Gal-9 siRNA or pcDNA. On transfection day 2, A172, U373MG, and U87MG cells were collected and analyzed for NLRP3 inflammasome by flow cytometry. (A) Representative histogram of glioma cell line (A172) with NLRP3 and caspase-1 knockdown or overexpression. (B–C) NLRP3 and caspase-1 expression in glioma cells subjected to siRNA or pcDNA was calculated by flow cytometry (n = 3). *P < 0.05; **P < 0.005l; ***P < 0.0005; paired t-test.

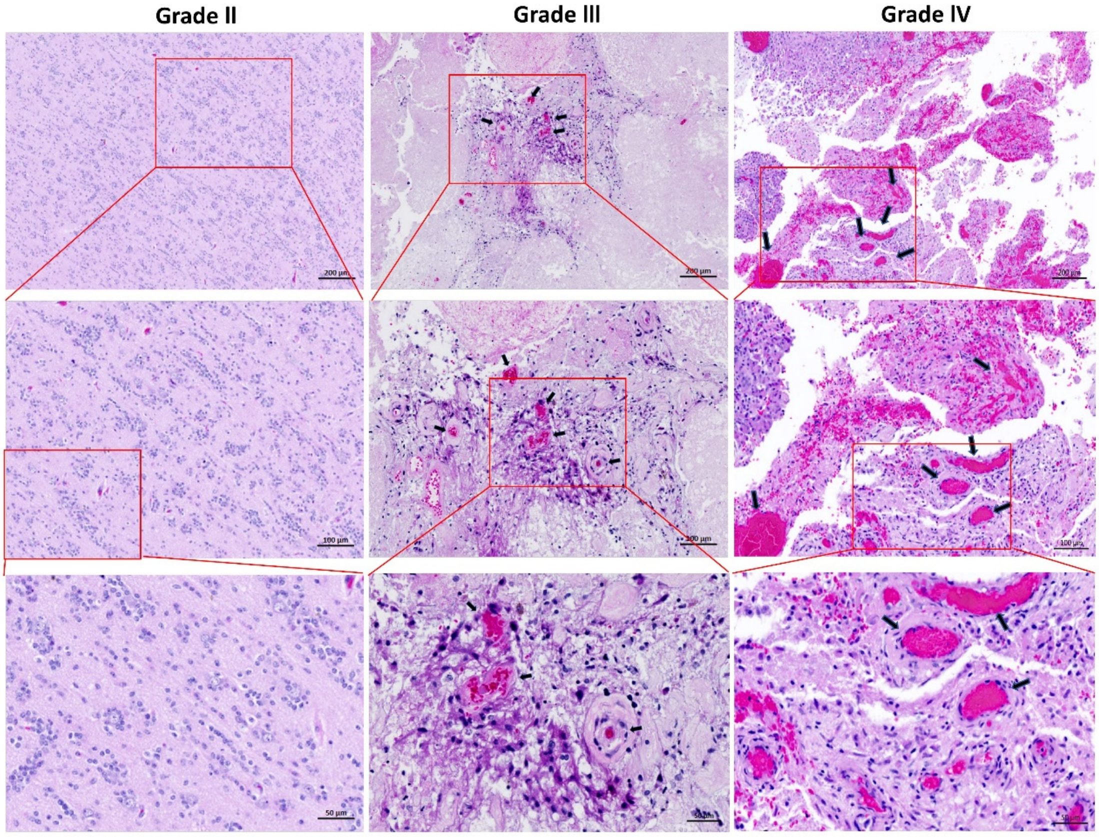

Supplementary Figure S10. Inflammatory microenvironment according to glioma malignancy. Each grade glioma (2, 3, and 4) subjects were stained with H&E reagent and analyzed with Zeiss LSM confocal microscopy (n = 9). Representative images were shown. Neo-capillaries were highlighted with black arrow.

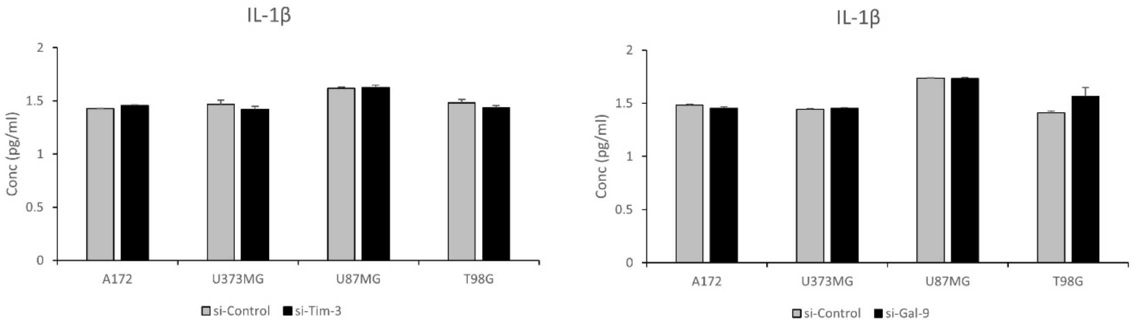

Supplementary Figure S11. IL-1β secretion did not significantly change in response to Tim-3/Gal-9. Glioma cells were pretreated with 50 nM siRNA control. Supernatant was collected on day 2 and IL-1β release was evaluated by ELISA according to kit manufacturer's instructions (n = 3).
